# How LGBTQ + adults’ experiences of multiple disadvantage impact upon their health and social care service pathways in the UK & Ireland: a scoping review

**DOI:** 10.1186/s12913-025-12232-8

**Published:** 2025-02-13

**Authors:** Mark Adley, Amy O’Donnell, Stephanie Scott

**Affiliations:** Population Health Sciences Institute, Faculty of Medical Science Newcastle University, Baddiley-Clark Building, Richardson Road, Newcastle, NE2 4AX UK

**Keywords:** Evidence synthesis, Healthcare access, LGBT*, Multiple disadvantage, Scoping review, UK, Ireland

## Abstract

**Background:**

Despite increased awareness of the significant health and healthcare inequalities experienced by minoritised groups, limited research considers the interaction of multiple domains of social disadvantage. This review therefore sought to explore how LGBTQ + adults’ experiences of homelessness, substance use, and criminal justice involvement impact upon their access to and use of health and social care services in the UK and Ireland.

**Methods:**

A qualitative scoping review was conducted in accordance with the PRISMA-ScR framework. Electronic database and web searches identified 26 eligible peer-reviewed and grey literature documents published between 2010–2024. The data were charted, coded, and knowledge gaps identified.

**Results:**

Data were coded thematically, clustered around the concept of normativity. Descriptive qualitative techniques were applied to explore how this was enacted and experienced. Synthesis across the literature identified experiences of discrimination and anticipated stigma that acted as barriers to accessing and engaging with services.

**Conclusions:**

Structural normativity and the privilege afforded to hegemonic population groups impacted upon this population’s access to and use of services. The review adds depth and context to questions around the lack of visibility or engagement in services by LGBTQ + people with experience of disadvantage, and contributes to the wider literature on improving service access for marginalised, underserved, or disadvantaged communities.

**Supplementary Information:**

The online version contains supplementary material available at 10.1186/s12913-025-12232-8.

## Terminology

Within the current study the term LGBTQ + is used to refer to people who are marginalised along axes of sexual orientation and/or gender identity, and who are (although not exclusively): lesbian, gay, bisexual, transgender, queer/genderqueer, questioning, intersex, agender, asexual, pansexual. This paper adheres to SAGER guidelines around sex (in biological terms) and gender (in cultural terms) [1]. Specific cultural terminology and acronyms are included in the list of abbreviations at the end of this article.

## Background

In 2012, Duncan’s [2] review of key texts within the field of severe and multiple disadvantage concluded that *‘further research is called for on groups and issues not covered by available literature: [including] lesbian, gay, bisexual and transgender (LGBT) people with complex needs’* (pp. 13–14). Research in the UK and Ireland has tended to focus on LGBTQ + people along single axes of disadvantage, such as experiences of homelessness [3], substance use [4], or offending [5]. That the authors of this thesis are aware of, studies specifically exploring LGBTQ + multiple disadvantage have been limited to a single grey literature report [6]. There have been ongoing calls for research around LGBTQ + severe and multiple disadvantage [7], and for research that *‘account[s] for compounding effects of multiple forms of marginalization within and in addition to SGM (sexual and gender minority) identities’* [8].

Given the paucity of information about severe and multiple disadvantage in LGBTQ + populations, the scoping review methodology was selected for this review for its potential to deliver a high-level summary of a relatively unexplored topic area [9] and to identify key factors related to this concept [10]. Rather than necessarily describing research findings in any detail, as in a systematic review, scoping reviews can be conducted in subject areas in which there are knowledge gaps, or where concepts require clarification [11]. Arksey and O'Malley (12) also acknowledge the benefits of a broad sweep across the published and grey literature that can make the scoping review *‘a useful way of mapping fields of study where it is difficult to visualize the range of material that might be available… and disseminating research findings to policy makers, practitioners and consumers who might otherwise lack time or resources to undertake such work themselves’*.

An initial sweep of the grey literature revealed comments such as the following, that hinted at some of the challenges of conducting research on the topic of LGBTQ + multiple disadvantage:

*‘people from the LGBTQ*+ *community tend to be under-represented amongst people accessing support services…’ *[13]

*‘our local MD (multiple disadvantage) data highlights gaps in data on people from LGBTQ*+ *groups, which means we know less about their specific experiences and needs, with the risk that these are not included in future service plans’ *[14]*.*

Thus, if population data around severe and multiple disadvantage are drawn from services designed to support people experiencing multiple disadvantage, it is important to identity key factors relating to why LGBTQ + people who face multiple disadvantage might not access these services.

### Aims and objectives

The aim of a literature review is to identify what is and what isn’t known about its topic, to present a summary and synthesis of the relevant literature relating to the focus of the review question, and to relate the study to the larger, ongoing dialogue in the literature [15]. The literature review for the current study therefore aims to add background and context to this issue of the access to and use of services by LGBTQ + people who experience severe and multiple disadvantage. This scoping review seeks to answer the question: *How do LGBTQ* + *adults’ experiences of homelessness, substance use, and offending impact upon their access to and use of health and social care services in the UK and Ireland?* Its objectives are:

1. To use scoping review methods to identify and explore the existing published research regarding the intersection of sexual orientation and/or gender identity with experiences of homelessness, substance use, and offending.
2. To identify themes within the broader literature, using the evidence base derived from objective 1, with a view to synthesising the existing knowledge thematically and identifying research gaps.

## Methods

This scoping review is reported according to the PRISMA-ScR reporting checklist [16], included in Additional file 1.

### Protocol and registration

The protocol was registered on the Open Science Framework [17] on 17/07/2024 [18]. The protocol was drafted on 06/05/2024 after literature searches had been completed, but before selection of sources of evidence. Deviations from the published protocol from 06/05/2024 onwards can be found in Additional file 2.

### Eligibility criteria

The review adopted the Population, Concept, and Context framework [19] to construct eligibility criteria and objectives, supplemented by amended definitions of the three primary domains of severe and multiple disadvantage (SMD3) used by Bramley, Fitzpatrick (20). These are defined below in Table 1, with detailed eligibility criteria in Additional file 3.

**Table 1:** Eligibility criteria

**Population**	Concept	Context
LGBTQ + individuals or groups (defined by belonging to sexual orientation and gender identity minority groups) over the age of 18, with experience of multiple disadvantage	Consideration of access to and/or use of health and social care services	Studies conducted in the UK and Ireland
**The primary domains of severe and multiple disadvantage (SMD3)** amended from [20]		
Homelessness: a broad definition of homelessness is adopted, including not only rough sleeping, but also other forms of highly insecure and inappropriate accommodation		
Substance use: use of drugs or alcohol that requires treatment or support from services		
Offending: whether in custody or under supervision, as a result of multiple and/or non-trivial criminal convictions		

### Information sources

#### Published literature

To identify potentially relevant published literature, Ovid Medline, SCOPUS, and CINAHL electronic databases were searched. These three databases were selected to best address this paper’s subject area which bridges disciplines of health, population health, and social sciences. Database search strategies were initially drafted in tandem with library staff within the research team’s host university. Search terms for LGBTQ + populations created by Lee, Ylioja [21] were amended by the lead author to include recent terms such as *agender, pansexual,* and *non-binary.* A validated geographical search filter for the UK [22] was amended by the lead author to include Irish cities, based upon census data [23]. Database searches were conducted on 13/04/2024, supplemented by web searches on 10/05/2024 using Google Scholar [24].

#### Grey literature

##### Theses

The EThOS database [25] consists of UK theses dating back to 1925, that was downloaded on 01/05/2024.

##### Voluntary, Community, and Faith Sector (VCFS) and government documents

Web searches for grey literature took place between 01/05/2024 and 06/05/2024. Follow-up contact with an author was required for one of the VCFS reports, with clarification provided in an email from the report's author M. Moncrieff (monty@londonfriend.org.uk) on 25/03/2024. 

#### Additional material

Additional published and grey literature identified in web searches and citation lists were integrated into the sources of evidence.

### Search

#### Published literature

The database search strategy used search terms for: LGBTQ + populations, SMD3 categories (experience of homelessness, substance use, and offending), use of and access to health and social care services, and studies from UK and Ireland from the last 14 years to capture research from the onset of austerity measures implemented since 2010 [116]. The Medline search syntax for these terms is included in Additional file 4. Web searches used Google Scholar, with syntax included in Table 2.

**Table 2:** Google Scholar search syntax for published literature

	**Search terms**
**Homelessness**	“UK” OR “United Kingdom" OR Ireland AND LGBT* AND homeless* AND service* OR treatment
**Substance use**	“UK” OR “United Kingdom" OR Ireland AND LGBT* AND drug OR alcohol OR “substance use” AND service* OR treatment
**Offending**	“UK” OR “United Kingdom" OR Ireland AND LGBT* AND prison* OR probation OR offend* OR “criminal justice” AND service* OR treatment

The symbol * is used in Google searches as a wildcard matching any word or phrase

#### Grey literature

The EThOS database was opened in Microsoft Excel (639,253 items). Theses dated prior to 2010 were removed, leaving 277,168 items. Excel’s Advanced Filter was used to identify titles and abstracts containing keywords (Table 3). Web search syntax for VCFS and government documents are included in Additional file 5 and Additional file 6 respectively.

**Table 3:** Terms used in Excel’s Advanced Filter

	Excel: Advanced Filter search terms	
	LGBTQ + populations	SMD3 categories
**Search terms**	LGB* or *lesbian* or *gay* or *bisexual* or *transgender* or *queer* or *agender* or *non-binary* or *gender non-conforming*	*homeless* or *drug* or *alcohol* or *substance* or *prison* or *crime* or *offending* or *police* or *probation*

The symbol * is used in Google searches as a wildcard matching any word or phrase

#### Web search strategy

With no ‘gold standard’ for rigorous systematic grey literature search methods [26] the review followed recommendations by Briscoe, Nunns [27] including experimenting with different search engines, search terms, and advanced search features. Once an optimal method had been identified this was applied in a structured manner across the areas of investigation. Organisations’ websites were then searched manually for relevant documents using sites’ search functions or by searching manually. As government websites (e.g. *gov.uk*) did not respond to Boolean or wildcard search operands, Google’s advanced features were used to search the web with results restricted by domain (e.g. *site:.gov.uk*). Searches were conducted from google.co.uk, apart from google.ie (Republic of Ireland). Results were limited to the first five eligible reports or organisational websites for each of the specified countries, as not only are relevant documents more likely to be identified towards the beginning of search results, but researchers must also consider time and resource constraints [28].

#### Search engine and browser selection

After testing search results from Google Search, Google Scholar [29], and Mednar [30], Google Search returned the results most relevant to this review. Aiming to minimise the impact of personalised search histories, searches were conducted on a computer that had not previously been used for personal browsing, and a web browser (Chrome) [31] that had not been previously used on that computer. Searches were completed while in ‘guest’ mode (i.e. not connected to any Google accounts) and Incognito mode [32], with browser settings amended to delete its cache when all windows were closed [33]. Offers of customised search results and cookies were declined, and while the effectiveness of such approaches is disputed [34], unlike Google searches conducted on other devices, searches returned no ads or ‘sponsored’ listings.

### Selection of sources of evidence

#### Published literature

The lead author (MA) selected published literature, with screening conducted by MA and the co-authors (AOD and SS). MA and AOD double-screened titles and abstracts independently for inclusion. Where there was disagreement on inclusion of articles, further discussion took place between MA, AOD, and SS. Full-text documents were then retrieved for the remaining papers, which were double-screened independently by MA and SS.

#### Grey literature

The lead author selected and screened grey literature. When the relevancy of an article was not easily determined, this was discussed with AOD and SS.

### Data charting process

The data charting framework was developed by the lead author. As recommended by Peters, Marnie (36), it was developed iteratively and evolved to capture new data items of relevance to the review question.

#### Data management

All documents were imported into the data analysis software programme MAXQDA [36], and charting variables created manually. MAXQDA’s Summary Grid feature supported further charting of issues such as key theories and critiques.

### Data items

The following data were extracted from article characteristics:

- Document details
- Literature type
- Study aim (either directly or indirectly stated)
- Study location
- Primary domain
- Study population, sample size, and methods
- Data sources (separated into workstreams)
- Domains of social inequalities (e.g. ethnicity, gender, sexual orientation)

### Critical appraisal of sources of evidence

It is not necessary to complete an appraisal of the sources included in a scoping review, which instead seeks *‘to develop a comprehensive overview of the evidence rather than a quantitative or qualitative synthesis of data’* [35].

### Synthesis of results

An exploratory, thematic analysis approach was used to encode qualitative information, with a flexible and open approach to coding conducted in a heuristic, iterative manner [37]. Its approach was predominantly inductive, with overarching themes generated using Boyatzis’ [38] technique of ‘clustering’: organising themes in the context of other themes, hierarchically. This approach was selected for its relevance, as the majority of the codes were grouped around the underlying concept of *Normativity enacted within SMD3 services.* The second overarching theme was clustered hierarchically under this concept and encompassed *The impact of enacted normativity.*

## Results

### Selection of sources of evidence

**Fig. 1:**
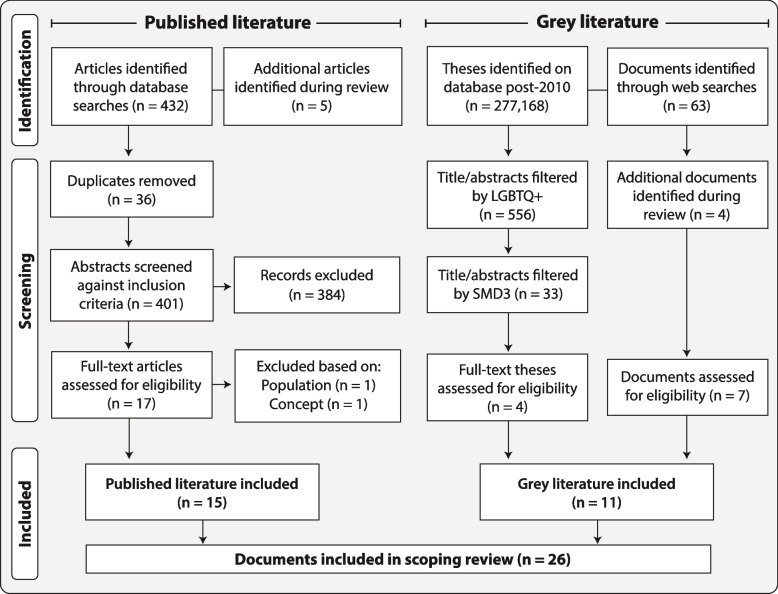
PRISMA flowchart

The number of published literature articles and grey literature documents screened at each level is shown in the PRISMA flowchart below (Fig. 1).

#### Published literature

A total of 432 papers were retrieved during database searches alongside additional articles (*n* = 5). After duplicates were removed the remaining 401 titles and abstracts were imported into the web-based software tool Rayyan [39] for screening. Full-text documents were retrieved for the remaining 17 published articles, with 15 meeting inclusion criteria.

#### Grey literature

Of the 639,253 theses in the EThOS database, 277,168 were dated 2010 or later. Titles and abstracts were searched using Excel’s Advanced Filter to identify references to LGBTQ + populations (*n* = 556), and then SMD3 populations (*n* = 33), which were then manually searched by MA. Six theses were selected for screening of full texts: one was ineligible for inclusion, one had no full text available, and the remaining four were included. Searches of VCFS and government websites identified 63 documents, with four additional documents identified during the review process. Of these 67 documents, seven were assessed as eligible for inclusion.

#### Characteristics of sources of evidence

The 26 documents included in this review are presented in the data tables in Additional file 7, in which the following additional acronyms are used: MSM (Men who have sex with men), WSW (Women who have sex with women), TGNC (Transgender and gender non-conforming, i.e. gender identity, as distinct from sexual orientation).

### Results of individual sources of evidence

The review presents basic descriptive analysis such as frequency counts, while also aiming for a clear, visual presentation [35, 40]. Of the 26 included studies, 25/26 were primary research and 1/26 was a discussion paper that also contained qualitative data. The earliest report was published in 2012, and 16/26 were from the last five years (2019–2024). Additional detail of the data charted within the review is provided in the tables and figures within this section. Table 4 shows the focus of the included studies by i) primary population, ii) primary LGBTQ + population, and iii) primary domain of disadvantage.

**Table 4:** Focus of scoping review studies: populations and domains

**Focus of studies: populations and domains (*n* = 26)**	n		%
**Primary population**			
LGBTQ +	23		88.4
Professionals	3		11.5
**Primary LGBTQ + population (grouped by category)**			
LGBTQ +	14		53.8
MSM only	7		26.9
TGNC only	5		19.2
**Primary domain of disadvantage**			
Substance use	11		42.3
Offending	8		30.8
Homelessness	6		23.1
Multiple	1		3.8

**Fig. 2:**
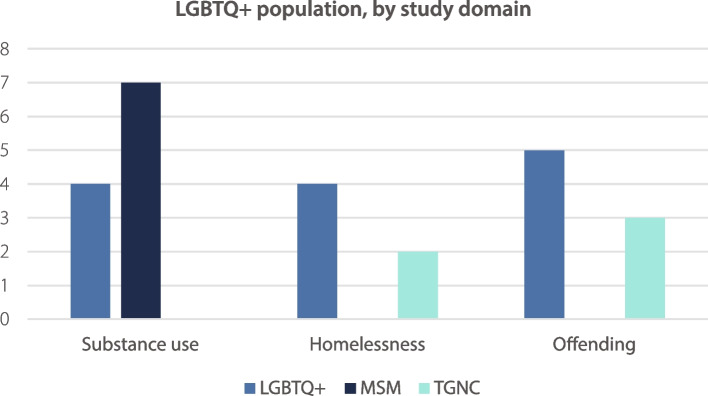
LGBTQ + population within study domains

Fig. 2 shows LGBTQ + population studied within each domain. Twenty-five studies were included (the study containing multiple domains was omitted), with data in text form as follows:

- Substance use: LGBTQ + the primary population in 4/11 (36.4%), MSM in 7/11 (63.6%), and TGNC in 0/11 (0%)
- Homelessness: LGBTQ + the primary population in 4/6 (66.7%), MSM in 0/6 (0%), and TGNC in 2/6 (33.3%)
- Offending: LGBTQ + the primary population in 5/8 (62.5%), MSM in 0/8 (0%), and TGNC in 3/8 (37.5%).

**Fig. 3:**
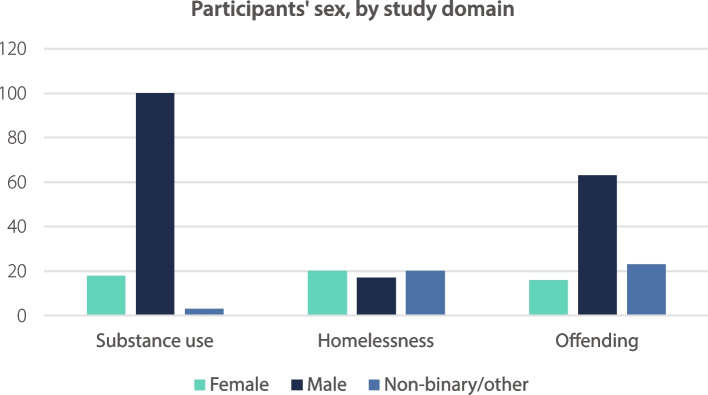
Participants’ sex, by study domain

Participants’ sex was grouped into three categories: female, male, and non-binary/other. Of the 23 studies with LGBTQ + people, 13/23 (56.5%) provided demographic data on participants’ sex, with 12/23 (52.2%) providing data which could be grouped into these categories. Fig. 3 shows the number of participants within each of the domains by sex, with data in text form as follows:

- LGBTQ + substance use (*n* = 121): female 18 (14.9%), male 100 (82.6%), non-binary/other 3 (2.5%)
- LGBTQ + homelessness (*n* = 57): female 20 (35.1%), male 17 (29.8%), non-binary/other 20 (35.1%)
- LGBTQ + offending (*n* = 102): female 16 (15.7%), male 63 (61.8%), non-binary/other 23 (22.5%).

**Fig. 4:**
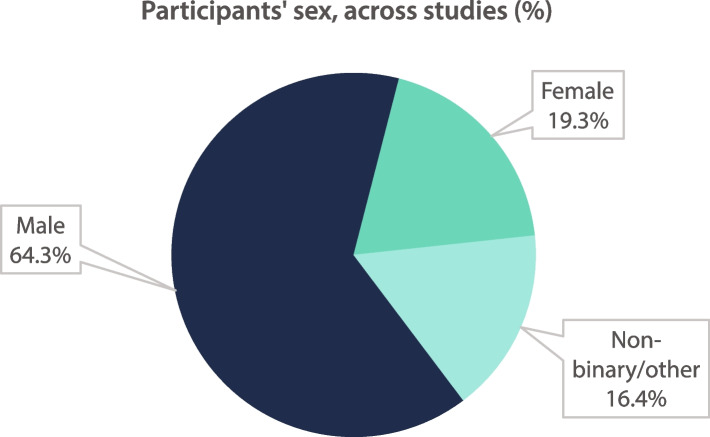
Participants’ sex, across studies (%)

Fig.4 shows that, within literature that included demographic data on sex that could be grouped as follows (*n* = 280) the breakdown was: female 54 (19.3%), male 180 (64.3%), Non-binary/other 46 (16.4%). Within studies that differentiated between transgender females and transgender males (*n* = 7), transgender females numbered 15/20 (75.0%) and transgender males 5/20 (25.0%).

**Fig. 5:**
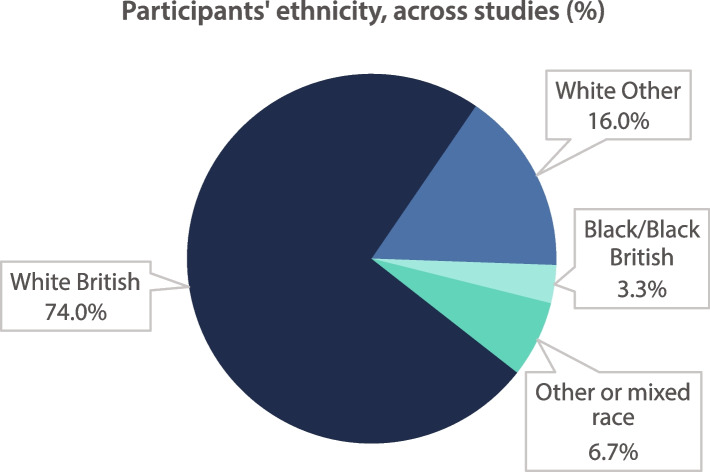
Participants’ ethnicity across studies (%)

Of the 23 studies with LGBTQ + people, 7/23 (30.4%) provided demographic data on participants’ ethnicity. For purposes of clarity these were grouped into four categories and are shown in Fig. 5. Totalled across the three domains (*n* = 150), the breakdown by ethnicity was: White British 111/150 (74.0%), White Other 24/150 (16.0%), Black/Black British 5/150, (3.3%), Other or mixed race 10/150 (6.7%). No participants of Asian or Asian British ethnicity were noted within studies that provided demographic data on ethnicity.

Of the 23 studies with LGBTQ + people, 4/23 (17.4%) contained data on employment and education, however it was not feasible to group or standardise this data due to inconsistent categories and missing data. Deprivation data was available for 1/23 (4.3%) of the studies.

### Synthesis of results

The synthesis of results did not seek to provide definitive conclusions, and instead acknowledged that it was *‘subject to the authors’ judgment and creativity’* [35]. Descriptive qualitative techniques explored the findings, with coding of data to the concept of *normativity*: applied here as a broader term that refers to the ‘mythical norm’ of (male/white/heterosexual/cisgendered/financially stable/middle class/able-bodied/thin) privileged normativity [41, 42]. Normativity applied leverage across multiple levels of influence and was of specific relevance to the review question.

The importance of transparency within the reporting of data has been identified [16] which in this case acknowledged the influence of the lead author’s positionality and the intersectional, queer, feminist theoretical underpinnings of the wider doctoral thesis of which this review was a component. There is convergence within these three theories around the concept of normative privilege, with privilege described as *‘an invisible package of unearned assets [which] takes institutionalised and embedded forms’* [43]. Intersectionality seeks to explore the impact of normative privilege on people’s social locations, lived experiences, and health outcomes [44, 45], and intersectional, queer, and feminist standpoints all highlight the value of the perspective offered by those on the outside of normative privilege. Thus, although the findings were summarised qualitatively, the discussion also considered the quantitative data in this section. Responding to calls to *‘queery… institutions that (re)create heteronormativity’* [46], the review sought to question who was and wasn’t included within research, drawing from the charted demographic data (and/or lack of it). In doing so however, the authors acknowledge the subjectivity within the qualitative analysis of the review’s content, and recognise its descriptive nature.

## Themes

### Normativity enacted

#### Not one of ‘us’

Normative practices within SMD3 services positioned LGBTQ + people as not belonging within services' ‘normal’ remit. For example, heterosexist assumptions such as ‘*what does your husband do?’* immediately positioned LGBTQ + participants outside of the norm 'Mia', in [47]. This process was also mirrored at systemic levels. Across the network of services this resulted in LGBTQ + people with experience of disadvantage being ‘cycled’ between organisations without receiving tangible support, resulting in *‘inappropriate, repeated use of resources’* [48]. One pattern identified across the three SMD domains was of LGBTQ + people being moved on to other services for more ‘specialist’ care, for example:

*‘Staff reported that they would signpost individuals ​[engaging in chemsex] to external organizations they felt might be more appropriate’ *[49]*.*

Sometimes there was a lack of consideration of the appropriateness of these onward referrals, such as a doctor referring their patient to an organisation that no longer existed [50]. In both homeless shelters and prisons, the actions of staff suggested that those who were outside of ‘the norm’ were causing problems for those who were in positions of normative privilege. England (52) noted how, in situations where conflict arose within homeless service provision, trans people in Wales were *‘consistently moved out of ​the space, and subject to enhanced surveillance’* (p. 844). This ‘othering of the problem’ was repeated within prison settings. After reporting that they had been sexually assaulted, LGBTQ + people were removed from their wings and placed into segregation units [52, 53]. While this may have been presented as being for their own protection, being moved into segregation is viewed as punitive and was experienced as punishment by LGBTQ + people, rather than acts of care [54, 55]. Additionally, being separated from main wings, either by being placed in segregation or onto ‘vulnerable prisoner’ wings, can reduce people’s access to prison programmes and support services [56].

#### A lack of cultural awareness

When services did not address the topics of sexual orientation and/or gender identity with clients, this was often perceived as lack of care for, or consideration of the impact that these had on people’s lives. A client of an alcohol treatment service in Scotland describes how this issue was ‘brushed over’ by their worker, even after they had raised it as an issue of concern:

*‘I said I think this might be… a big link as to why (I was drinking). ​But they didn’t actually ask very much about it… ‘Ben’, in *[47]*.*

LGBTQ + people reported feeling uncomfortable about whether to raise the topic of their sexual orientation and/or gender themselves [47], and when they did mention this, there were instances of staff directing them elsewhere on the grounds that they lacked understanding of needs specific to LGBTQ + communities [57]. Staff often missed key signals from applicants about sexual orientation and gender identity and failed to consider the danger faced by many LGBTQ + people, such as hate crime and domestic violence going unrecognised or being minimised [57, 58]. This was often interpreted as a lack of care and consideration for people’s ‘non-normative’ issues.

#### An inequitable impact

Policies that were applied generally, at times had an inequitable impact upon LGBTQ + populations. For example, while the ‘local connection’ tests implemented by English and Welsh housing systems can be an issue for everyone seeking local authority housing, for LGBTQ + people it might mean having to move away from places with LGBTQ-friendly services into areas with less welcoming or understanding services [59]. Similarly, systemic issues such as overcrowding in prisons can have an additional significance for LGBTQ + people who can additionally *‘be targets of stigma, derision and deviance, leading to feelings of ​​helplessness, isolation and humiliation’* [53]. For gay male prisoners in Ireland hiding their sexual orientation out of fear of assault and violence, the close quarters of shared cell accommodation carried with it the additional fear of this being noticed by cell-mates [55]. Normative processes and assumptions that are enacted within policy and practice might thus fail to consider their impact upon the access to and use of services by LGBTQ + people facing multiple disadvantage.

### Normativity experienced

#### Internalisation

Those who experience multiple disadvantage often bear shame and stigma associated with ‘*dislocation from societal norms*’ [20]. For many LGBTQ + participants this was exacerbated by stigma related to their dislocation from heterosexual or cisgendered norms. Internalised stigma influenced help-seeking behaviours Milburn (61), with participants referring to the additional shame and embarrassment that ​​they felt in connection with their trans identity and identity as ‘an alcoholic’:

*‘I’ve always been ashamed of who I am… (that) I was transgender… (and) I was an alcoholic… It’s the stigma’ ‘Ash', in *[47]*.*

#### Anticipation

Also acting as a barrier to accessing help was a lack of trust in health and social care services, expectations of institutional discrimination [6, 61], and fears of being judged by professionals [62, 63]. Participants avoided services because of concerns over mainstream services being oriented to White, straight men [64], the lack of privacy in hostels, and risks of experiencing homophobia and transphobia from other service users [65]. Such concerns were to some extent borne out by professionals’ comments. Temporary accommodation providers believed that their hostels were ‘LGBT-friendly’ while acknowledging that LGBT residents experienced sexual harassment, physical and verbal aggression, threatening behaviour, and harassment from fellow residents on a regular basis [66]. Although it may not be fair to assign meaning from these quotes due to their limited context, such attitudes might suggest a general acceptance of, or perhaps resignation to LGBTQ + discrimination within their services, rather than a desire to challenge the source of discriminatory behaviour.

#### Hiding

Hiding one’s LGBTQ + identity was a common theme across the SMD3 domains. This was perhaps most evident within prison settings, where disclosure could result in reprisals and violence [54, 55]. For many participants, their experiences of homelessness, substance use, and the criminal justice system involved assessing the risk to themselves and their bodies in being themselves within services [6, 58].

#### Avoidance

In the face of discrimination, whether enacted or anticipated, LGBTQ + participants avoided accessing support: *‘How am I going to go and tell him [my doctor] I am gay and all that? So forget about that, I’m not going’* ‘Participant 6’, in [60]. Transphobic and homophobic bullying between residents in homeless services was a key driver of people avoiding service provision [65]. In one prison establishment, Harris (55) noted that the LGBT group was held in a room in the middle of the wing, and that in order to attend it people would have to present staff with a movement slip. Attending the group would therefore risk disclosing people’s identity, and LGBTQ + prisoners therefore felt unable to attend the group. Thus, without considering the stigma, discrimination, and threats of violence faced by LGBTQ + people experiencing multiple disadvantage, services can exclude them.

#### Dodging

LGBTQ + participants ‘dodged’ mainstream services; finding alternative ways of managing their health and social care needs to avoid discrimination, stigma, or lack of consideration of their specific needs [6]. The majority of participants in one study [67], when asked about their harm reduction needs related to chemsex use, preferred to visit a sexual health service. LGBTQ + people experiencing homelessness also dodged mainstream services, finding alternatives such as sofa-surfing or sleeping in cars preferable to accessing statutory housing support [66].

#### Comparison

Relative perceptions of disadvantage impacted upon service use and access, with LGBTQ + participants comparing their experiences of disadvantage with that of ‘normative’ SMD3 clients. Participants described their needs as not being severe enough to warrant seeking assistance or a place in services [60, 63] and minimised the seriousness of their experiences, believing for example that squatting and sofa-surfing were not ‘real’ homelessness [65]. That some people’s experiences of severe and multiple disadvantage were prioritised over others’ was also reinforced by professionals:

*‘I think [substance use] services, we’re so geared up to alcohol and OCUs [opiate and crack cocaine users], aren’t we? So, that’s our bread and butter. So, that’s what we’re dealing with all the time, whereas chemsex, it isn’t…’* ‘N04’, in [49].

#### When things goes well

Bryan and Mayock [68] noted the importance of researchers concerned with social justice countering negative discourse, and contributing instead to the politics of progress. Specialist LGBTQ + services and organisations providing advocacy support were described in positive terms [66], while partnership work and innovative, flexible approaches to service promotion and delivery such as pop-up clinics and outreach were found to be effective [69]. Some participants favoured support from LGBTQ + staff who shared their gender identity [63], or their sexual orientation and gender [58]. For others it was support from LGBTQ + staff with lived experience of disadvantage that was most valuable [6]. However, the sexual orientation and/or gender identity of staff was not always an issue, for example in the prison estate where positive relationships with any staff *‘can facilitate prisoners seeking support’* [54].

## Discussion

### The essential/intersectional axis

The acronym LGBTQ + contains a diverse mix of minoritised sexual orientations and/or gender identities, however it risks masking this complexity. This scoping review identified reports of systems, services, and staff essentialising people along these lines, with LGBTQ + identities pathologized: presumed to be the ‘reason for’ people presenting at SMD3 services. Examples of this included people’s transgender identities pathologized by homelessness services [57] and by mental health services [6, 60]. One participant shared how his probation worker’s immediate response to finding out he had attended the LGBT support group in the prison had been to treat his sexuality as a risk factor and ask questions about his sex life in and outside prison [54]. A queer theoretical approach asks us to trouble the heteronormativity of these events, which reinforce the irregularity or deviance of ‘others’ [70]. As noted by Ristock and Julien [71]: *‘These same lines of inquiry are not asked of heterosexuality’.*

This essentialising often masked people’s other, individual needs. Review findings supported intersectionality’s position that the impact of stigma may be contextually bound [72]; thus when an LGBTQ + person presents at a service for support with their homelessness, the stigma related to their homeless identity may supersede that of their LGBTQ + identity [47]. Research within the prison estate noted how the reinforcement of masculine heterosexist norms placed gay men at increased risk of assaults and violence [55]. Elsewhere the hegemony of the heterosexual, cisgendered male and his privileged position within SMD3 services impacted on the discrimination experienced by those ‘others’ [52, 53]. England [51] argued that the provision of homeless services prioritised male primacy, and noted that:

*‘a conflict exists between a service-mandate to provide care to particularly vulnerable (presumed) cis straight men and to protect groups impacted by enactments of cis male dominance’* (p. 838).

With greater divergence from norms came greater discrimination. For example transgender women were at greater risk of discrimination from staff in prison settings than ‘masculine-presenting’ gay men, who were more closely aligned to social norms and therefore presented less of a problem to normative service provision [52]. However, alternative perspectives came from prison staff sensitive to the needs of transgender female prisoners [73] who framed this in terms not of discrimination, but of the degree of adaptation required to existing workplace processes. Regardless of one’s perspective, the ‘other’ was perceived to be the problem.

LGBTQ + participants’ experiences within services could be conceptualised along an essentialist-intersectional axis, which at one end pathologized their sexual orientation and/or gender identity, and at the other acknowledged the impact of wider systems of power on their access to and use of services. For example, the majority of the literature on LGBTQ + homelessness focused on young people as opposed to adults. This emphasis framed homelessness as a response to issues faced by young people such as bullying, or family conflict or rejection [74], in doing so minimising how LGBTQ + people are affected by the same structural issues that increase the risk of homeless generally such as poverty, racism, sexism, domestic violence, or sexual abuse. For LGBTQ + adults these issues may have additional consequences, intersecting with minoritised sexual orientation and/or gender identities in distinct ways [7, 75–77].

The data charted in this review highlighted that within the included studies on LGBTQ + substance use the majority of the literature focused around MSM and chemsex use. This is aligned with the wider literature, such as the review which noted that *‘the amount of research focusing on drug use among gay men and/or MSM far outweighs the amount of research on drug use among other members of the LGBT community’* [61]*.* Without dismissing the importance of this research, this scoping review allowed for reflection on the hegemony of this MSM group within LGBTQ + substance use research, and its position along essentialist-intersectional lines. The setting of this research within sexual health services is a matter of consideration, as was the significant amount of literature excluded from this review that associated MSM and chemsex use with risks and treatment of sexually transmitted infections. Such discussions might reinforce wider discourse associating gay and bisexual men with sexually transmitted infections, HIV/AIDS, and promiscuity, and may further confound sexual orientation with sexual activity [78, 79]. It also leads to questions about the preponderance of research funding allocated to chemsex use in MSM populations: whether this has the wellbeing of this population at its core, or concerns about the risk to the ‘wider’ (read heteronormative) public health, as believed to be the case at the start of the HIV/AIDS pandemic [80].

Examination of what, or who, was missing from the extant literature also informed the interpretation of its results. It was noted that the prevalence of chemsex in non-MSM groups was poorly understood, with specialist services described as being *‘exclusively designed for cisgender MSM’* [48]. Women’s health in general is underfunded compared to that of men’s [81], and within that, lesbians and bisexual women are frequently subsumed within ‘wider’ LGBTQ + research [82]. Aligned with this review’s findings was the observation that WSW engaging in sexualised drug use had been largely neglected from previous research (52). Although sexualised drug use and associated risks were common in WSW this was only when a wide range of drugs were considered, as opposed to the drugs that define chemsex [83]. Therefore, the sexualised drug use of WSW is rendered invisible within a framework which defines chemsex by the drugs used by MSM. Troubling this definition of chemsex leads to questions around its prioritisation within the study of LGBTQ + substance use research, and whether this might reflect a greater importance placed on the health of (sexual minority) men over (sexual minority) women.

The issue of ethnicity was also widely neglected. Within the seven studies that provided demographic data on participants’ ethnicity, two papers noted the lack of ethnic diversity within their studies and recommended further research into the interaction of different marginalised identities [47, 50]. Three studies made no mention of ethnicity outside of demographic tables and introductions [64, 67, 84], while one [63] mentioned only that the lack of ethnic diversity in their study *‘is not wholly surprising, given the under-representation of ethnically minoritised groups in substance use research’.* Notably, of the 377 study participants identified within these seven studies, not one participant identified as Asian or Asian British. Only one study out of the seven, a doctoral thesis exploring the barriers to mental health services from an LGBTQ + perspective, took specific steps to recruit people seeking asylum or mentioned race and religion in interviews [60]. There is an emerging literature from the UK around the often-complex intersections of sexual orientation and/or gender identity with ethnicity, religion, and community [85, 86], including the specific harm faced by LGBTQ + people of colour [87]. This had broadly failed to be recognised or acknowledged within the included literature.

While intersectionality is often conceptualised simply in terms of identity categories this does it a disservice, rendering invisible concepts such as privilege and the structures of oppression, and intersectionality’s potential for converging around a politics of solidarity [88, 89]. The relative scarcity of focus upon sexual minority women and LGBTQ + people of colour within this review's literature may reflect existing social privilege, the social invisibility of women’s needs, and further disadvantage and marginalisation experienced at the intersection of structural racism, sexism, and socioeconomic inequalities [90, 91]. Mandviwala, Hall [92] write of the *culture of exclusion* that privileges White, Christian, heteronormative men. Research within this scoping review suggested how normative privilege, especially that which was enacted along patriarchal lines, served to create a culture of exclusion within services for LGBTQ + people facing multiple disadvantage [49, 51, 54, 56–58].

Analysis of participant demographics within this review’s literature identified the most prevalent demographic was (gay) male, White British, and cisgendered. While this scoping review does not make any claims of generalisation, its analysis leads to further consideration of the extent to which wider societal privileges might be enacted within the myriad social positions contained within the LGBTQ + acronym, and the potential impact of these privileges upon ‘others’. If, as suggested by this review’s findings, the privileged position of the White British, cisgendered male in wider society is mirrored within SMD3 services, and the White British, cisgendered gay male occupies a privileged position within LGBTQ + people with experience of multiple disadvantage, this raises further questions about the experiences of those outside of this hegemonic group, and their ability to access and engage with health and social care services.

### Cycle of (SMD3) invisibility

While this review has brought attention to the impact of normativity within and across SMD3 services, questions still remain. The recent English and Welsh census data reported that of people identified as homeless, more than double identified as lesbian, gay, bisexual, or ‘other/LGB + ’ (7.7%) than in the general population of England and Wales (3.2%) [93]. Despite such visibility within census data however this population remains largely hidden. Academics working within LGBTQ + homelessness noted challenges in recruitment [58], having to amend their language and thus redefine SMD for it to appear valid to LGBTQ + participants. Elsewhere, LGBTQ + people dismissed their own experiences as not matching up to their own preconceptions of disadvantage, and those held by SMD3 services [49, 50, 59]. This resonates with the following quote from the sole piece of research exploring LGBTQ + SMD in the UK:

*‘This frame was not sufficient to understand the full range of [LGBT] people’s experiences, and did not capture the different kinds of marginalisation they had faced’* [6]*.*

The ‘cycle of invisibility’ experienced by marginalised groups was identified in legal settings in the 1980s [94]. Later research [95] identified this cycle within a heteronormative educational environment in which discrimination was permitted and people’s concerns went unnoticed or ignored, leading to an unsafe environment for LGBTQ + people. Within the provision of LGBTQ + aged care services [96, 97], service providers assumed they didn’t have any LGBTQ + clients, and therefore that they did not need to consider their needs in future service provision.

While this scoping review is broadly aligned with these existing frameworks, its perspective of LGBTQ + disadvantage offers additional depth and context. Avoidance, anticipation, and dodging of services are described in depth. Workplace cultures and staff attitudes also featured within LGBTQ+ SMD3 invisibility. Jokes and ‘banter’ stigmatised LGBTQ + participants who did not feel empowered to challenge these [54, 56, 98], not only because this banter was normalised and widely accepted, but also because discrimination went unchallenged by staff who were perceived to collude with it by their lack of action [51–53].

There is also the impact of anti-discrimination legislation to consider. Zago (57) suggested that such legislation creates moments of visibility and invisibility, requiring organisations to attend momentarily to LGBTQ + populations within their services for reporting purposes. This could be self-defeating in settings such as prisons, where disclosing one’s LGBTQ + identity could lead to verbal and physical abuse, intimidation, and sexual assault [54, 98] thereby resulting in apparently low numbers of LGBTQ + people [52]. England (58) also highlighted how, despite its intentions, such legislation could inadvertently operate as a distancing mechanism. She suggested that it could lead to resistance from staff and services and, given the polarised debates around trans people’s rights to access services at the time of writing this review, that a commitment to supporting anti-discrimination legislation could be framed *‘as a political resistive act’* (p. 381). Thus, staff who aligned themselves with anti-discrimination legislation could be perceived as taking a political stand within this debate, as opposed to supporting anti-discrimination policies in more general terms. Resistance from staff towards anti-discrimination policies might thus translate into workplace cultures in which normativity is reinforced.

The review raises further questions around access to specialist services. For example, while the majority of MSM interviewed had access to specialist substance use support, specialist provision in the UK and Ireland is generally limited to cities with large LGBTQ + populations such as Manchester, Brighton, Dublin, and London [48, 50, 62, 67, 69, 84]. Much less is known about the access to or use of mainstream, commissioned services by those outside of these ‘gaybourhoods’.

Despite recommendations within the wider body of literature that sexual orientation and/or gender identity should generally be discussed and its data widely collected [99, 100], this was not standard practice across the review. This is a nuanced topic however, and may not simply be omission on the part of researchers. For example, qualitative studies generally recruit smaller numbers of participants than those expected of quantitative studies, and some researchers chose not to report demographic data to protect participant confidentiality [53, 58]. Cultural issues were also a factor, for example England (52) did not routinely ask participants about their gender identity, as *‘to require this was felt to risk reinscribing a normative, categorical approach to gender identity which particularly erases identities which are not static or binarised’*. Hankivsky’s article exploring intersectionality scholarship noted *‘the limitations of research that emphasizes pre-determined classifications’* [101]. While this is also aligned with queer theory’s deconstructive approach to socially-constructed identity categories, as illustrated by the exclusion of England’s article from this review’s charting of demographic data, doing so can potentially render invisible specific, important, cultural identities.

The cycle of invisibility model describes how a lack of data collection can contribute to marginalised groups’ invisibility becoming embedded into services. Low apparent numbers are assumed to represent both a low service need, and no need to adjust services to accommodate for specific cultural needs. Accordingly, normative service provision is reinforced [102]. Within this scoping review, normativity was seen to impact upon the access to and use of services by LGBTQ + adults with experiences of homelessness, substance use, and offending. Facing or anticipating stigma, exclusion, and violence upon disclosure of their LGBTQ + identities, some hid their identities, some disclosed them and were referred elsewhere, while others found alternatives or avoided mainstream services altogether. Professionals brushed aside clients’ sexual orientation and/or gender identity, or simply assumed them to be heterosexual or cisgender. The topic of invisibility is thus entwined in this review’s response to the question of how LGBTQ + adults’ experiences of homelessness, substance use, and offending impact upon their access to and use of health and social care services in the UK and Ireland.

#### Limitations of the scoping review process

Although the review benefited from the structure and rigour afforded by adherence to the PRISMA-ScR checklist, a single author charted and extracted data whereas elsewhere it has been recommended that multiple authors should be involved in these processes [35, 103]. Also open to methodological criticism was the grouping of some of the demographic data. While this attempted to bring cohesion and a degree of clarity to a diverse mix of categories, the process involved merging, for example transgender men and women into a single category which potentially masked both sex and gender differences. Claims of cultural insensitivity could also be targeted at the review’s adherence to fixed definitions of sex, and the decision to omit the study that merged female sex and gender into a single category [63] from charting of demographic data.

A limited scope of health and social care services were considered within the review. Although the access to and use of general healthcare services was discussed, many of the documents focused on the same SMD3 service area as the study’s population. Thus, people using substances widely discussed access to substance use services, and people experiencing homelessness discussed access to housing services. Only one study [60] broke this mould and explored the barriers to mental health services from the perspective of LGBTQ + people facing homelessness. Overall, the review’s limited consideration of multiple health and social care services may have narrowed its findings.

The review’s web search syntax may also have restricted results. Whereas the Medline search syntax for LGBTQ + populations consisted of 53 lines of search terms, internet searches used only the single term LGBT*. This may have failed to capture documents that instead used terms such as sexual orientation and gender identity, or had as their focus specific populations such as lesbians or transgender people.

The definition of adulthood was another issue of consideration. In some settings, notably substance use and criminal justice, adults were defined as aged 18 + while in others (particularly within homeless provision) as 16 and over. Where it was not possible to separate the data for people aged under 18, a number of key studies were excluded that may have broadened the scope of the review’s findings [74, 104, 105]. The study’s eligibility criteria may also have benefited from the inclusion of government policy [12].

Although the review aspired to be relevant to other countries, there may be limitations to its geographical transferability. Terminology may confound the issue: while the phrase ‘multiple disadvantage’ is one that has been adopted in the UK and Ireland, the overwhelming majority of research around this topic area is conducted within the US and Canada and rarely uses this term. Additionally, the scoping review comes from a perspective of countries with a generous welfare state provision relative to other countries, which may further limit its relevance. In countries without state-provided healthcare the concept of ‘access to healthcare’ is positioned very differently than in the UK and Ireland. Similarly, in countries where being perceived to be using drugs can lead to custodial confinement in facilities operated by police or military staff [106], receiving substandard care within a community drug treatment service that provides free medical supervision might be viewed as a blessing.

As with all research syntheses, it is important to acknowledge that this review is inherently influenced by multiple layers of interpretation and representation. Suri [107] explored the ethics of this issue, noting that research synthesis involves at least three layers of interpretation or representation: by participants in the primary study, by authors who interpreted their evidence, and then again by the research synthesist. She also considered the *anticipatory* influences of stakeholders. Within the current review for example, issues were omitted from analysis in anticipation of the responses that they might receive, and their potential for controversy. An additional influence to take into account was that as this scoping review was written as part of the lead author’s doctoral study, there will have been bias towards data that fit within the wider narrative of the thesis.

## Conclusions

Despite these limitations, this scoping review explored gaps in the literature within this field of study, and in addition to contributing to the UK and Irish perspective may also add context elsewhere. This is perhaps timely, given that research emerging from the US and Canada during the final stages of writing this review is attending to intersectionality within health settings e.g. [108, 109].

In response to the question of how LGBTQ + adults’ experiences of homelessness, substance use, and offending impact upon their access to and use of health and social care services in the UK and Ireland, the review identified patterns across the SMD3 domains. By exploring structural normativity and the primacy of population groups it shone a light on wider systems of privilege, and their influence on this population’s access to and use of services. Potentially of cross-cultural significance, the review’s findings contribute to the wider literature on improving service access for marginalised, underserved, or disadvantaged communities [110, 111].

Methodologically, there were benefits from both the structure and flexibility afforded by a systematic scoping review. For example, as cross-cutting themes were identified, analytical and charting processes were swiftly adapted to integrate consideration of population demographics. At the same time as it was flexible, adherence to the PRISMA-ScR checklist added rigour to the review process.

Khalil and colleagues reinforced the exploratory rather than explanatory nature of scoping reviews, and cautioned against *‘[the] tendency for reviewers to want to write the conclusion and recommendations section of their [scoping] review as one would for a systematic review’* [40]. Definitive conclusions cannot be drawn from this scoping review that, by its very nature, lacks an assessment of the quality of the included literature or its risk of bias. However, exploring the question at the heart of this review has potentially shone a light on areas of normative privilege, and their influence, that may previously have been hidden from view.

There is limited research that captures the health and social care experiences of LGBTQ + people who have faced multiple disadvantage, with recent recommendations for approaches that embrace *‘how the intersection of multiple marginalized identities shape health experiences’* [108]. This review has identified specific gaps in the literature around the experiences both of LGBTQ + people of colour, and sexual and gender minority women, who have experienced disadvantage. Ralphs and Gray (70) posited that ‘*the focus needs to shift to addressing the problem of non-engagement’,* and the invisibility and exclusion of participants within this scoping review support this statement. Given the importance of relationships with staff on participants’ engagement with services, studies that included the experiences of both LGBTQ + people and professionals added depth and context to this issue. Further research might also consider the dominance of the White British male within LGBTQ + research, and the experiences of LGBTQ + people outside of main urban ‘gaybourhoods’ where specialist services are not available and commissioned services the only option.

## Supplementary Information

Additional file 1.

Additional file 2. [112, 113].

Additional file 3. [114–117].

Additional file 4.

Additional file 5.

Additional file 6.

Additional file 7.

## Data Availability

All data generated or analysed during this study are included in this published article [and its supplementary information files]. The datasets generated and/or analysed during the current study are also available as a Supplementary Table in the Open Science Framework, within this project: https://osf.io/fd4zm/.

## Abbreviations

AIDS: Acquired Immune Deficiency Syndrome
BBV: Blood-borne virus
Cisgender or Cis: A person whose sense of gender identity (socially/culturally constructed) corresponds with their sex (biological sex, or sex assigned at birth)
Heteronormativity: A worldview that privileges heterosexuality as the natural and normal orientation, which forms the basis for many social structures and norms
HIV: Human Immunodeficiency Virus
LGBTQ+: Within the current study the term LGBTQ+ is used to refer to people who are marginalised along axes of sexual orientation and/or gender identity, and who are (although not exclusively): lesbian, gay, bisexual, transgender, queer/genderqueer, questioning, intersex, agender, asexual, pansexual. This acronym was selected over others, such as LGBTQIA+, LGBTI, LBGTIQ, LGBTQ2S (2-spirit), LGBTGNG (gender non-conforming), as it is in common usage within the UK. In doing so, it acknowledges that this may exclude the explicit naming of certain groups internationally (such as 2-spirit, a term used within traditional Native American communities). Outside of global north and global western settings, the term LGBTQ+ may represent concepts of personal identity that are not universal, and the acronyms SOGI (sexual orientation and gender identity) or SOGIESC (sexual orientation, gender identity, gender expression, and sex characteristics) may be used instead (IOM. Training Aide – IOM SOGIESC Glossary of Terms. 2021. Available from: https://www.iom.int/sites/g/files/tmzbdl486/files/documents/Training-Aide-IOM-SOGIESC-Glossary-of-Terms.pdf)
LGB: Lesbian, gay, or bisexual (i.e. sexual orientation, as distinct from gender identity)
LGBT: Lesbian, gay, bisexual, or transgender. An earlier acronym, before the inclusion of the wider populations within LGBTQ+ (above)
MSM: Men who have sex with men
PRISMA-ScR: Preferred Reporting Items for Systematic reviews and Meta-Analyses extension for Scoping Reviews
SAGER: Sex and Gender Equity in Research
SGM: Sexual and gender minorities (an alternative term to LGBTQ+)
SMD: Severe and multiple disadvantage
SMD3: The three primary domains of severe and multiple disadvantage: experience of homelessness (including insecure accommodation), substance use (requiring treatment), and offending (in custody, or under supervision)
STD/STI: Sexually transmitted disease/infection
TGNC: Transgender and gender non-conforming (i.e. gender identity, as distinct from sexual orientation)
UK: United Kingdom
VCFS: Voluntary, Community, and Faith Sector: the organisational sector outside of statutory provision, elsewhere referred to as the VCSE (Voluntary, Community, and Social Enterprise) or Third Sector
WSW: Women who have sex with women
