# Supplementary material for: How LGBTQ + adults’ experiences of multiple disadvantage impact upon their health and social care service pathways in the UK & Ireland: a scoping review
Source: BMC Health Serv Res. 2025 Feb 13;25:244. doi: 10.1186/s12913-025-12232-8 (PMC11823026; doi:10.1186/s12913-025-12232-8)
Supplement: Supplementary file 2 — Additional file 2. [112, 113]. [file 12913_2025_12232_MOESM2_ESM.zip › Additional file 2 footnote_ESM.docx]

Additional file 2: footnote

1. Evans E, Lépinard É. Intersectionality in Feminist and Queer Movements: Confronting privileges. London, UK: Taylor & Francis; 2020.

2. Etherington C. Race, gender, and the resources that matter: An investigation of intersectionality and health. Women & Health. 2015;55(7):754-77.
