# Supplementary material for: How LGBTQ + adults’ experiences of multiple disadvantage impact upon their health and social care service pathways in the UK & Ireland: a scoping review
Source: BMC Health Serv Res. 2025 Feb 13;25:244. doi: 10.1186/s12913-025-12232-8 (PMC11823026; doi:10.1186/s12913-025-12232-8)
Supplement: Supplementary file 3 — Additional file 3. [114–117]. [file 12913_2025_12232_MOESM3_ESM.zip › Additional file 3 footnote_ESM.docx]

Additional file 3: footnote

1. Mak S, Thomas A. An Introduction to Scoping Reviews. Journal of Graduate Medical Education. 2022;14(5):561-4.

2. World Health Organisation. Maternal, newborn, child and adolescent health and ageing: Data portal. 2022. Available from: https://platform.who.int/data/maternal-newborn-child-adolescent-ageing/adolescent-data.

3. McKibbin WJ, Stoeckel A. Global Fiscal Consolidation, CAMA Working Paper No. 9/2011. Canberra, Australia; 2011. Available from: https://cama.crawford.anu.edu.au/pdf/working-papers/2011/092011.pdf.

4. McNeill S, O'Donovan D, Hart N. Access to healthcare for people experiencing homelessness in the UK and Ireland: a scoping review. BMC Health Services Research. 2022;22(1):910.
